# Changes in cortical activation during upright stance in individuals with chronic low back pain: An fNIRS study

**DOI:** 10.3389/fnhum.2023.1085831

**Published:** 2023-02-03

**Authors:** Yan Li, Zhaoqiang Xu, Hao Xie, Ruochen Fu, Wai Leung Ambrose Lo, Xue Cheng, Jiajia Yang, Le Ge, Quihua Yu, Chuhuai Wang

**Affiliations:** Department of Rehabilitation Medicine, The First Affiliated Hospital, Sun Yat-sen University, Guangzhou, Guangdong, China

**Keywords:** functional near-infrared spectroscopy, low back pain, dorsolateral prefrontal cortex, balance, motor dysfunction

## Abstract

**Introduction:**

Postural control deficits are a potential cause of persistent and recurrent pain in patients with chronic low back pain (CLBP). Although some studies have confirmed that the dorsolateral prefrontal cortex (DLPFC) contributes to pain regulation in CLBP, its role in the postural control of patients with CLBP remains unclear. Therefore, this study aimed to investigate the DLPFC activation of patients with CLBP and healthy controls under different upright stance task conditions.

**Methods:**

Twenty patients with CLBP (26.50 ± 2.48 years) and 20 healthy controls (25.75 ± 3.57 years) performed upright stance tasks under three conditions: Task-1 was static balance with eyes open; Task-2 was static balance with eyes closed; Task-3 involved dynamic balance on an unstable surface with eyes open. A wireless functional near-infrared spectroscopy (fNIRS) system measured cortical activity, including the bilateral DLPFC, pre-motor cortex (PMC) and supplementary motor area (SMA), the primary motor cortex (M1), the primary somatosensory cortex (S1), and a force platform measured balance parameters during upright stance.

**Results:**

The two-way repeated measures ANOVA results showed significant interaction in bilateral PMC/SMA activation. Moreover, patients with CLBP had significantly increased right DLPFC activation and higher sway 32 area and velocity than healthy controls during upright stance.

**Discussion:**

Our results imply that PMC/SMA and DLPFC maintain standing balance. The patients with CLBP have higher cortical activity and upright stance control deficits, which may indicate that the patients with CLBP have low neural efficiency and need more motor resources to maintain balance.

## Introduction

Low back pain (LBP) is becoming a growing socioeconomic burden (Hartvigsen et al., 2018; Cieza et al., 2021; Chiarotto and Koes, 2022) because it increases the risk of disability and contributes to poor mental health. Many studies have confirmed that pain-related disabilities and intensity were likely related to the altered structure and function of muscles in patients with CLBP (Emami et al., 2018; Yu et al., 2021). For example, when facing external disturbances or changes in sensory input, patients with CLBP show greater body sway velocity and area (della Volpe et al., 2006; Willigenburg et al., 2013; da Silva et al., 2018), delayed muscle activity (Knox et al., 2018) and altered spinal kinematics (Meier et al., 2019), which could accelerate spinal degeneration and muscle spasms, injuries, and fatigue. This consequence may lead to chronic dysfunction and recurrent pain (Panjabi, 1992). Therefore, low back pain and postural control deficits often interact. Understanding the mechanism of postural control deficits in patients with CLBP would help formulate appropriate CLBP rehabilitation strategies (Hodges and Moseley, 2003).

Existing studies have confirmed that postural control deficits in patients with CLBP may be related to functional and structural changes in the sensorimotor-related brain regions (Goossens et al., 2018). Neuroimaging studies have shown that both direct and indirect locomotor networks are responsible for postural control. The direct locomotor network consists of the primary motor cortex (M1) and the cerebellum. The indirect locomotor network consists of other cortical areas, such as the prefrontal cortex (PFC) and supplementary motor area (SMA) (Herold et al., 2017b). Functional magnetic resonance imaging (fMRI) studies showed that structural and functional changes in pain regulation-related brain regions commonly occurred in patients with CLBP, especially in the dorsolateral prefrontal cortex (DLPFC) (Fritz et al., 2016; Li et al., 2018). Findings from functional near-infrared spectroscopy (fNIRS) studies suggested that the DLPFC plays an important role in allocating attentional resources and working memory (Mihara et al., 2008; Fujita et al., 2016) during postural control tasks or walking in healthy young (Mihara et al., 2008) and elderly (Teo et al., 2018; Pelicioni et al., 2021) individuals, as well as in patients with neurological diseases such as Parkinson’s disease (Pelicioni et al., 2020). However, empirical evidence demonstrating the relationship between DLPFC dysfunction and postural control deficits in patients with CLBP is still lacking.

Functional changes in brain regions in the direct or indirect locomotor network are related to the postural control deficits of patients with CLBP (Goossens et al., 2018). For example, fMRI studies have shown that regional changes in gray and white matter (Kregel et al., 2015), together with resting-state functional connectivity in the primary somatosensory cortex (S1), SMA, M1, and cerebellum of patients with non-specific CLBP were decreased compared with healthy participants (Pijnenburg et al., 2015). Second, decreased functional connectivity between M1 and the cerebellum was significantly associated with poor balance performance in patients with CLBP (Pijnenburg et al., 2015). Evidence from transcranial magnetic stimulation (TMS) demonstrated cortical reorganization of trunk muscle representation in the motor cortex of patients with CLBP (Tsao et al., 2008; Li et al., 2021). An electroencephalogram (EEG) study illustrated a correlation between changed positive peak amplitudes and altered postural kinematics and muscle activity during posture perturbations in patients with CLBP (Jacobs et al., 2016). However, postural control is a dynamic process. Traditional neuroimaging methods, such as fMRI and TMS, impose significant physical constraints on mobility. fNIRS is a novel neuroimaging technology that can determine the concentrations of oxygenated hemoglobin and deoxyhemoglobin in the local cortex by detecting optical parameters, indirectly reflecting neural activity (Sakudo, 2016). It has the advantages of portability, non-invasive nature, and low sensitivity to motion artifacts during dynamic balance tasks (Teo et al., 2018; Maidan et al., 2022). Although fNIRS has a lower temporal resolution than EEG, it is suitable to measure brain activity during upright stance tasks in this study, which did not require high temporal resolution. However, few studies have used fNIRS with balance tasks in patients with CLBP.

The present study aimed to explore the cortical activation in the DLPFC of patients with CLBP during bipedal standing on a force platform under different upright stance conditions using fNIRS. We hypothesize that the patients with CLBP show upright stance control dysfunction and require increased DLPFC activation compared with healthy individuals.

## Materials and methods

### Participants

Twenty patients with CLBP (CLBP group) and 20 healthy controls (HC group) were enrolled in this study. All the participants were right-handed. The inclusion criteria for the CLBP group were as follows: (1) repeated low back pain lasting more than 3 months; (2) age range of 18–45 years; (3) visual analog score (VAS) greater than 3; (4) body mass index (BMI) within the normal range; (5) non-specific low back pain. The exclusion criteria for the CLBP group were as follows: (1) arthritis, tumor, neurological disease, fracture, intervertebral disk herniation, ankylosing spondylitis, rheumatism, and other diseases with clear causes; (2) other diseases that may lead to motor disorders, such as vestibular dysfunction; (3) pregnancy; (4) mental illness, such as depression disorder. The patients with mental illness were excluded according to the patient’s chief complaint, physical examination, imaging data, and other major medical data. Healthy controls were age-, sex-, height-, weight-, and BMI-matched. The exclusion criteria for healthy controls were acute and/or recurrent low back pain within the last 3 months and a history of chronic pain episodes.

### Instruments and experimental design

**FIGURE 1:**
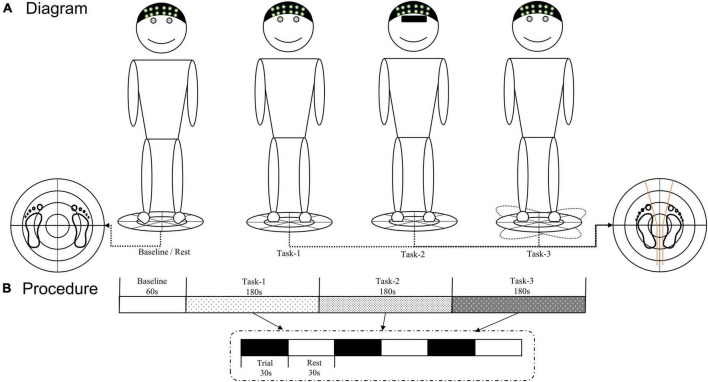
**(A)** Diagram of three upright stance tasks. **(B)** fNIRS testing procedure. The TecnoBody system was used to collect the COP signals of each participant during the upright stance control task, and fNIRS technology was used to detect real-time hemodynamic signals. COP, center of pressure; fNIRS, functional near-infrared spectroscopy.

The VAS was used to assess the degree of pain in patients with CLBP. Use the TecnoBody force platform (PK254P; TecnoBody, Bergamo, Italy) to test the center of pressure (COP) excursion and velocity to evaluate the participants’ upright stance stability during upright stance tasks. Sample all the COP signals at a rate of 100 Hz. The participants were required to stand barefoot on the platform with their arms resting at their sides. Ge et al. (2021b) described the standardized position of the foot on the platform. This system can provide a stable and movable platform by setting different parameters. Upright stance tasks for three conditions were evaluated (Figure 1): (Task-1) static balance on a stable surface with eyes open; (Task-2) static balance on a stable surface with eyes closed; (Task-3) dynamic balance on an unstable surface with eyes open. In our pilot study, we tried to do the upright stance on an unstable surface with eyes closed. However, we found that this condition could increase the risk of falling, and some patients with CLBP who had poor balance ability could not complete the upright stance on an unstable surface with their eyes closed. Therefore, the upright stance on an unstable surface with eyes closed was not involved in the task condition of our study.

**FIGURE 2:**
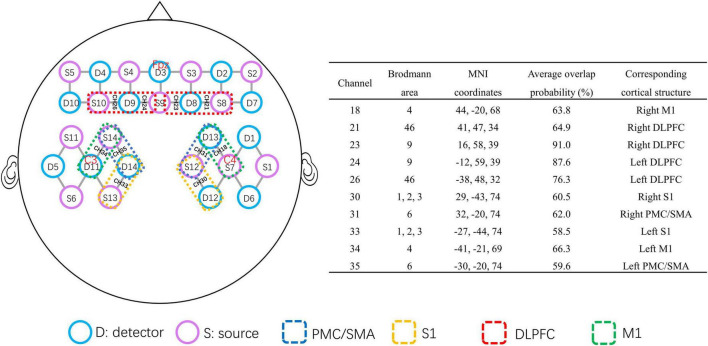
Brain map of the fNIRS head probe covering the prefrontal and parietal cortices. The purple circles represent near-infrared light source fibers. The blue circles represent detectors. The short gray lines represent the channels between the sources and detectors. The channels in the red dotted box represent the DLPFC. The channels in the blue dotted box represent the PMC/SMA. The channels in the green dotted box represent M1. The channels in the yellow dotted box cover S1. The MNI coordinates of the selected channel are provided in the right table. fNIRS, functional near-infrared spectroscopy; DLPFC, dorsolateral prefrontal cortex; M1, primary motor cortex; PMC/SMA, pre-motor cortex and supplementary motor area; S1, primary somatosensory cortex.

A multichannel fNIRS system (NirSmart-6000A, Danyang Huichuang Medical Equipment Co., Ltd., Jiangsu, China) was used to record changes in the concentrations of oxygenated hemoglobin [HbO] and deoxygenated hemoglobin [HbR] in the frontal and parietal regions. The wavelengths were set to 730 and 850 nm. Data were sampled at a frequency of 11 Hz. Thirty-five channels (defined as the midpoint of the corresponding light source-detector pair) were established, with 14 light sources and 14 detectors for measurement, which were positioned by referring to the standard international 10–20 system of electrode placement. D11 locate at the C3 position, S7 locate at the C4 position, and D3 locate at the Fpz position (Figure 2). The distance between the fNIRS source and detector was set to 3 cm. The probe locations (located at Nz, Cz, Al, Ar, Iz, referring to the standard international 10–20 system of electrode placement) were measured by an electromagnetic 3D digitizer device (Patriot, Polhemus, VT, USA) on a model head. Then the acquired grand-averaged coordinates were processed by NirSpace (Danyang Huichuang Medical Equipment Co., Ltd., Jiangsu, China) to estimate the Montreal Neurological Institute (MNI) coordinates and associated brain regions of channels together with the overlap probability of the channels (Tsuzuki et al., 2007). MNI coordinates refer to the coordinates obtained by the Monte Carlo simulation method to calculate the propagation path of light which are collected by 3D positioning and mapped. Each MNI coordinate is used as the sphere center (radius = 10 mm) to calculate the voxel percentage of each brain region covered in this sphere to the total voxels in the sphere as the overlap probability of brain region (Lancaster et al., 2007; Tsuzuki et al., 2007; Laird et al., 2010). Due to the near-infrared technology itself, the MNI coordinate as the center of the sphere does not necessarily fall in the center of the brain regions. The brain region with the highest probabilistic value underneath each fNIRS channel was identified (Figure 2).

Before the task began, the requirements and precautions were explained to the participants. Participants stood quietly on the force platform, wore a portable fNIRS head cap, and completed the corresponding tasks according to a voice prompt. Each participant completed three trials under each condition. Each trial lasted for 30 s with 30 s rest between trials. During the baseline (60 s) and rest periods, each participant was required to stand on the stable platform and look forward with their eyes open. During each trial of all the tasks, the position of the participants’ feet on the platform was standardized by using a V-shaped frame (Figure 1).

### Data processing

All COP signals were filtered at 8 Hz (by a 30th order low-pass FIR filter with zero phase) and down-sampled at 20 Hz. COP measurements included sway length (mm), sway area (mm^2^), anteroposterior (AP) velocity (mm/s), and mediolateral (ML) velocity (mm/s). The mean COP parameters for the three trials in each task were calculated for each participant.

The NirSpark (Danyang Huichuang Medical Equipment Co., Ltd., Jiangsu, China) software package was used to preprocess the fNIRS data. The raw fNIRS signal was first converted into changes in optical density by taking the logarithm of the signal. Next, noisy channels with a very low optical intensity were eliminated (coefficient of variation > 10%). Then, bandpass filters were used to remove the effects of instrumental noise and physiological components, with a cut-off frequency of 0.01 Hz for the high-pass filter and 0.1 Hz for the low-pass filter. Finally, the [HbO] and [HbR] concentration changes were obtained using the modified Beer-Lambert law (Cope et al., 1988).

The general linear model (GLM) Δ[Hbx] = X * β + ε is a robust statistical model used to detect cerebral cortex activity during tasks. Y is the change in [HbO] or [HbR] concentration. X is the predicted stimulation evoked responses generated by convolving the task onset with the canonical hemodynamic response function. β is the coefficient (weight) of that stimulus condition for that source-detector channel, and ε is the error term representing all noise in the recording. The regression coefficient, beta (β) value (μmol), reflects the activation degree of each channel (Dans et al., 2021; Zheng et al., 2021). The beta (β) value, as well as the standard errors of [HbO] and [HbR] for each channel, were estimated for each trial in both groups. The current study focuses on the analysis of [HbO], which was the most sensitive parameter for regional brain blood oxygenation. For the [HbO] signal, positive β values indicated increased task-related cortical activation, and negative β values indicated decreased task-related activation (Dans et al., 2021).

In this study, we selected the bilateral S1 (Brodmann area, BA1, 2, 3), M1 (BA4), DLPFC (BA9, BA46), and pre-motor cortex and supplementary motor area (PMC/SMA) (BA6) in the frontoparietal lobe as the regions of interest (ROIs). The right and left S1 were covered by channels 30 and 33, respectively; the right and left M1 by channels 18 and 34, respectively; the right and left DLPFC by channels 21 and 23, and channels 24 and 26, respectively; the right and left PMC/SMA were covered by channels 31 and 35, respectively (Figure 2). We performed group analysis based on the ROIs.

### Statistical analysis

All statistical analyses were performed using SPSS v26.0 (IBM Corp., Armonk, NY, USA). The level of significance was set at α = 0.05. The homogeneity of variances and normality of the distribution of the parameters were tested using Levene’s and Shapiro–Wilk tests, respectively. Descriptive statistics were used to describe demographics. Differences in age and BMI between the CLBP and HC groups were determined using an independent sample *t*-test. The difference in sex percentage between groups was determined using the Chi-square test.

The data of mean COP parameters were analyzed by repeated-measures analysis of variance (ANOVA) with condition (Task-1, Task-2, and Task-3) as the within-subject variable and group as a between-subject factor (CLBP group and HC group). For each ROI, we calculated the mean of the group-averaged β estimates across the channels of the ROI. The two-way repeated-measures ANOVA tested differences in cortical activation between groups under different conditions for each ROI. Pillai’s trace test was used. *Post hoc* pairwise comparisons with Bonferroni adjustment were applied for significant main or interaction effects. *T*-tests were used to determine whether the regression coefficients (β) were statistically non-zero. The Benjamin-Hochberg correction procedure was used to adjust the *p*-value in the *t*-test at the group level based on the GLM model to decrease the false discovery rate (FDR) (Benjamini and Hochberg, 1995).

## Results

### Demographic data

Table 1 summarizes the characteristics of the participants in the two groups. The statistical results showed no significant differences between the two groups in age, height, weight, body mass index, and sex ratio.

**TABLE 1:** Demographic and clinical data of CLBP and HC groups.

Variable	CLBP (*N* = 20)	HC (*N* = 20)	CLBP *vs.* HC
Sex (male/female)	6/14	8/12	*χ^2^*_(1)_ = 0.440, *p* = 0.507
Age (years)	26.50 ± 2.48	25.75 ± 3.57	*t*_(19)_ = 0.772, *p* = 0.445
Height (m)	1.67 ± 0.09	1.66 ± 0.07	*t*_(19)_ = 0.223, *p* = 0.826
Weight (kg)	61.60 ± 10.70	58.25 ± 8.97	*t*_(19)_ = 0.917, *p* = 0.370
BMI	22.01 ± 1.83	21.05 ± 2.28	*t*_(19)_ = 1.318, *p* = 0.203
VAS	5.17 ± 1.56	N/A	N/A

Data are mean ± SD. BMI, body mass index; CLBP, chronic lower back pain group; HC, healthy control group; SD, standard deviation; VAS, visual analog scale.

### Upright stance control performance

**FIGURE 3:**
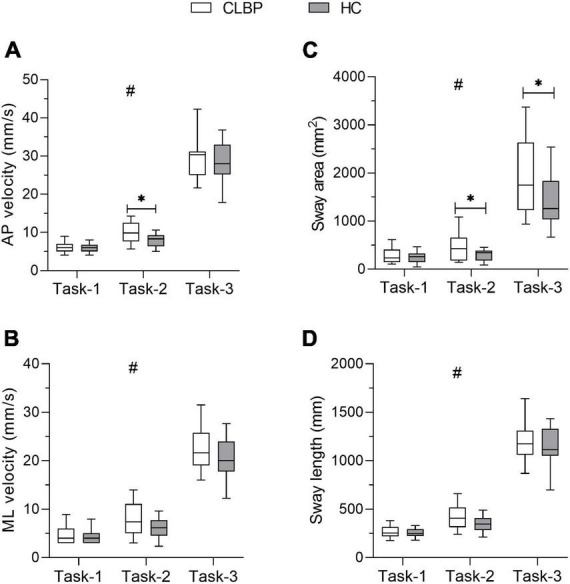
COP paraments for each group under three upright stance conditions. The box plot shows the median and the 25th and 75th quartiles of **(A)** AP velocity, **(B)** ML velocity, **(C)** sway area, and **(D)** sway length. Lower and upper error lines display the 5th and 95th percentiles. *Represents the interaction between group and condition, *p < 0.05; ^#^represents the main effect of condition. CLBP, chronic lower back pain group; HC, healthy control group; AP velocity, anteroposterior velocity; ML velocity, mediolateral velocity.

The four COP parameters under the three conditions for the two groups are listed in Table 2 and Figures 3A–D. Significant group effects were found in the ML velocity and sway area, demonstrating that the CLBP group had higher ML velocity and sway area than the HC group (*ps* < 0.05) (Table 2). The main effects of the condition were found for four COP parameters (Table 2). The results of *Post hoc* analysis indicated that participants presented larger COP parameters in Task-2 and Task-3 compared with Task-1, and COP parameters in Task-3 were larger than in Task-2 (*ps* < 0.001) (Figures 3A–D). The ANOVA showed a significant interaction of group × condition for the AP velocity and sway area (Table 2). *Post hoc* analysis showed that the CLBP group had a larger AP velocity than the HC group during Task-2 (*p* = 0.013) (Figure 3A). And the CLBP group had a larger sway area than the HC group during Task-2 (*p* = 0.037) and Task-3 (*p* = 0.016) (Figure 3C), but not in Task-1.

**TABLE 2:** Results of the repeated-measures ANOVA using the fixed factors “Condition” (Task-1, Task-2, and Task-3) and “Group” (CLBP group and HC group) for four COP parameters.

COP parameters	Effect		*Df*	*F*	*P* value	*η^2^p*
AP velocity	Main	Group	1, 38	2.956	0.094	0.072
	Condition	2, 37	525.990	<0.001***	0.966
	Interaction		2, 37	3.860	0.030*	0.173
ML velocity	Main	Group	1, 38	5.742	0.022*	0.131
		Condition	2, 37	279.947	<0.001***	0.938
	Interaction		2, 37	3.091	0.057	0.143
Sway area	Main	Group	1, 38	8.567	0.006**	0.184
		Condition	2, 37	91.922	<0.001***	0.832
	Interaction		2, 37	4.264	0.022*	0.187
Sway length	Main	Group	1, 38	2.290	0.138	0.057
		Condition	2, 37	578.527	<0.001***	0.969
	Interaction		2, 37	2.626	0.086	0.124

*p < 0.05, **p < 0.01, ***p < 0.001.

ANOVA, analysis of variance; COP, center of pressure; AP, anteroposterior; ML, mediolateral.

### fNIRS results

**FIGURE 4:**
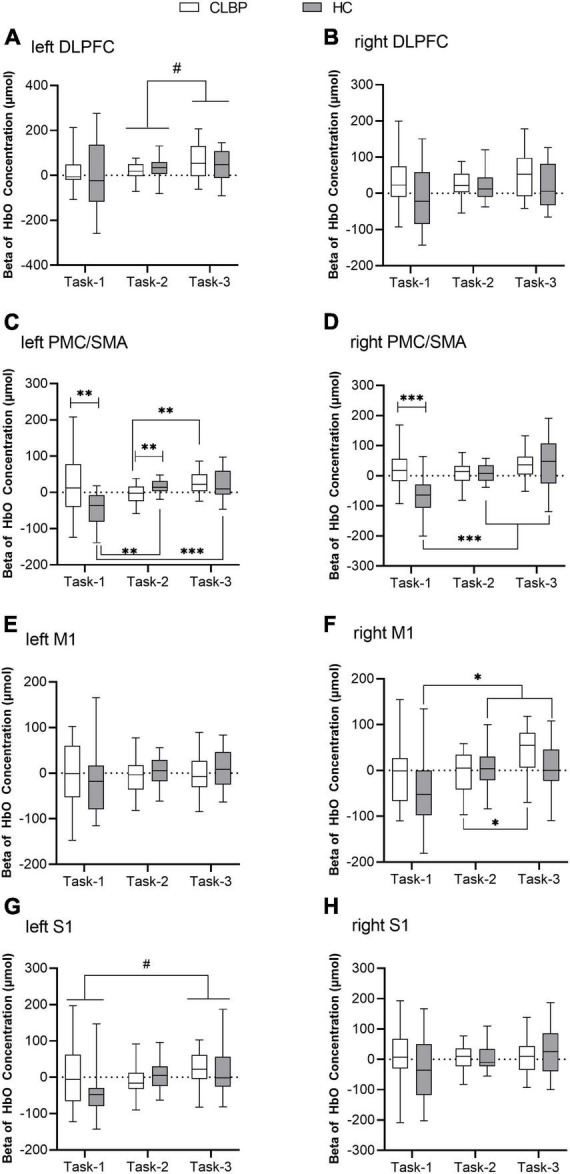
Beta values of [HbO] concentration in eight ROIs of two groups under three conditions **(A–H)**. **(A)** Left DLPFC; **(B)** right DLPFC; **(C)** left PMC/SMA; **(D)** right PMC/SMA; **(E)** left M1; **(F)** right M1; **(G)** left S1; and **(H)** right S1. The box plot shows the median and the 25 and 75th quartiles of beta value of [HbO] concentration. Lower and upper error lines display the 5 and 95th percentiles. *Represents the interaction between group and condition, *p < 0.05, **p < 0.01, and ***p < 0.001; ^#^represents the main effect of condition, ^#^*p* < 0.05. DLPFC, dorsolateral prefrontal cortex; fNIRS, functional near-infrared spectroscopy; M1, primary motor cortex; PMC/SMA, pre-motor cortex and supplementary motor area; S1, primary somatosensory cortex.

The results of the two-way repeated measures ANOVA are presented in Table 3 and Figures 4A–H. Group effects were observed in the right DLPFC and bilateral PMC/SMA (*ps* < 0.050) (Table 3). In right M1, the group effect was only a little short of significance (*p* = 0.050). The CLBP group had significantly greater bilateral PMC/SMA and right DLPFC activation than the HC group. Significant main effects of the condition were also found in the left DLPFC and right M1(*ps* < 0.050) (Table 3). The left DLPFC exhibited increased activation in Task-3 compared with Task-2 (*p* < 0.05) (Figure 4A). However, no significant interaction effect in bilateral DLPFC (Figures 4A, B). No significant between-group effect and interaction effect in bilateral S1 (Figures 4G, H), but a significant condition effect was found in left S1. The left S1 exhibited increased activation in Task-3 compared with Task-1 (*p* = 0.025) (Figure 4G). Statistical results also showed significant interaction effects of group × condition for bilateral PMC/SMA and right M1 (*ps* < 0.050) (Table 3). *Post-hoc* tests showed that the CLBP group had increased bilateral PMC/SMA activation compared with the HC group during Task-1 (*ps* < 0.01) (Figures 4C, D). In Task-2, the CLBP group had decreased left PMC/SMA activation compared with the HC group (*p* < 0.01) (Figure 4C). The CLBP group showed increased left PMC/SMA and right M1 activation in Task-3 compared with Task-2 (*ps* < 0.05) (Figures 4C, F), while no left PMC/SMA and right M1 activation differences between Task-1 and other tasks (*ps* > 0.05). At the same time, the HC group presented increased bilateral PMC/SMA and right M1 activation in Task-2 and Task-3 compared with Task-1 (*ps* < 0.05) (Figures 4C, D, F). There were no significant main effects and interaction effect in left M1 (Figure 4E).

**TABLE 3:** Results of the repeated-measures ANOVA using the fixed factors “Condition” (Task-1, Task-2, and Task-3) and “Group” (CLBP group and HC group) for each ROI.

ROIs	Effect		*Df*	*F*	*P* value	*η^2^p*
L-DLPFC	Main	Group	1, 38	0.092	0.763	0.002
		Condition	2, 37	3.905	0.029*	0.174
	Interaction		2, 37	1.095	0.345	0.056
R-DLPFC	Main	Group	1, 38	7.259	0.010*	0.160
		Condition	2, 37	1.527	0.231	0.076
	Interaction		2, 37	1.676	0.201	0.083
L-PMC/SMA	Main	Group	1, 38	5.377	0.026*	0.124
		Condition	2, 37	7.958	0.001**	0.301
	Interaction		2, 37	7.397	0.002**	0.286
R-PMC/SMA	Main	Group	1, 38	5.154	0.029*	0.119
		Condition	2, 37	9.633	< 0.001***	0.342
	Interaction		2, 37	9.358	< 0.001***	0.336
L-M1	Main	Group	1, 38	0.122	0.729	0.003
		Condition	2, 37	1.280	0.290	0.065
	Interaction		2, 37	0.783	0.465	0.041
R-M1	Main	Group	1, 38	4.091	0.050	0.097
		Condition	2, 37	5.359	0.009**	0.225
	Interaction		2, 37	3.261	0.050*	0.150
L-S1	Main	Group	1, 38	1.568	0.218	0.040
		Condition	2, 37	4.547	0.017*	0.197
	Interaction		2, 37	2.943	0.065	0.137
R-S1	Main	Group	1, 38	0.888	0.352	0.023
		Condition	2, 37	3.014	0.061	0.140
	Interaction		2, 37	2.037	0.145	0.099

*p < 0.05, **p < 0.01, ***p < 0.001.

ROIs, regions of interest; L, left; R, right; DLPFC, dorsolateral prefrontal cortex; M1, primary motor cortex; PMC/SMA, pre-motor cortex and supplementary motor area; S1, primary somatosensory cortex.

The *t*-test results of the group level based on the GLM are shown in Table 4. In Task-2, channel-21 (CH-21) (right DLPFC) in the CLBP group and CH-24 (left DLPFC) in the HC group were significantly activated. In Task-3, CH-18 (right M1), CH-21 (right DLPFC), CH-23 (right DLPFC), CH-24 (left DLPFC), CH-26 (left DLPFC), CH-31 (right PMC/SMA), and CH-35 (left PMC/SMA) in the CLBP group were significantly activated. Additionally, in Task-1, the CH-31 (right PMC/SMA) level in the HC group was significantly decreased.

**TABLE 4:** Significantly activated cortical regions during three tasks in CLBP group and HC group.

		CH-18 (R-M1)	CH-21 (R-DLPFC)	CH-23 (R-DLPFC)	CH-24 (L-DLPFC)	CH-26 (L-DLPFC)	CH-30 (R-S1)	CH-31 (R-PMC/SMA)	CH33 (L-S1)	CH-34 (L-M1)	CH-35 (L-PMC/SMA)
CLBP	Task-1										
	Task-2		2.959*								
	Task-3	3.357*	3.172*	3.478*	3.12*	3.535*		3.164*			4.183*
HC	Task-1							-3.269*			
	Task-2				3.705*						
	Task-3										

Data are t-values, t: statistical value of t-test; *p < 0.05, false discovery rate corrected. CH, channel; CLBP, chronic low back pain group; HC, healthy controls; L, left; R, right; DLPFC, dorsolateral prefrontal cortex; M1, primary motor cortex; PMC/SMA, pre-motor cortex and supplementary motor area; S1, primary somatosensory cortex.

### Correlation between cortical activation and upright stance control performance

In the CLBP group, under three conditions, there was no significant correlation between the VAS and the activity of ROIs (range of *r* = −0.389 to 0.337, range of *p* = 0.090–0.962). Only under Task-3 was the VAS positively correlated with the COP sway area (*r* = 0.474, *p* = 0.035). At the same time, right DLPFC activation positively correlated with COP sway length (*r* = 0.524, *p* = 0.018) and AP velocity (*r* = 0.519, *p* = 0.019).

In the HC group, under Task-1, ML velocity was negatively correlated with left PMC/SMA activation (*r* = −0.479, *p* = 0.033). Under Task-2, sway area was positively correlated with left DLPFC (*r* = 0.538, *p* = 0.014), right PMC/SMA (*r* = 0.551, *p* = 0.012) and left S1 activation (*r* = 0.567, *p* = 0.009). Under Task-3, the correlation between the sway area and left PMC/SMA activation was not currently significant (*r* = 0.428, *p* = 0.060).

## Discussion

This study is the first to examine the cerebral cortex activity in patients with CLBP during upright stance tasks through fNIRS. We report some new findings. We observed that patients with CLBP had significantly higher right DLPFC activation during upright stance tasks. Secondly, when standing still with eyes open, the patients with CLBP had increased bilateral PMC/SMA activation. Additionally, when visual feedback was absent, the patients with CLBP had decreased left PMC/SMA activation. Finally, the results also showed that PMC/SMA and DLPFC activation correlate with COP parameters.

A systematic review reported that elderly individuals with LBP had greater sway area and sway velocity when manipulating sensory inputs (Ge et al., 2021a). The patients with CLBP we recruited were young people. We still observed that they had larger sway area and sway velocity, which suggests that, compared with healthy controls, the patients with CLBP showed greater dependence on visual input (Mann et al., 2010; Lee et al., 2012) and dysfunction of proprioception (Mok et al., 2004; della Volpe et al., 2006) in maintaining balance. The correlation results also showed a positive relationship between pain intensity and sway area when dampening proprioceptive information. This result may further confirm the relationship between pain intensity and dysfunction in proprioceptive processing in patients with CLBP (Sipko and Kuczyński, 2013). A study reported that patients with CLBP showed greater AP velocity when standing on an unstable surface (della Volpe et al., 2006). However, our results did not show a significant difference between the patients with CLBP and healthy controls regarding AP velocity. The potential reason is that standing on an unstable surface was accompanied by limited visual feedback in their study, meaning fewer sensory information inputs and presenting participants with more significant upright stance challenges. Many factors, such as demography and methodological design, would contribute to variations in COP outcomes (MacRae et al., 2018). The choice of outcome indicators and the duration and repetition of the trials would help increase the reliability of the data.

Results showed a significant group difference in right DLPFC activation. Compared with healthy controls, the patients with CLBP had a higher right DLPFC activation during upright stance. This finding was supported by previous studies. In previous studies, higher DLPFC activation was observed in the elderly during walking compared with healthy younger individuals (Hawkins et al., 2018; Nóbrega-Sousa et al., 2020). According to the compensation-related utilization of neural circuits hypothesis (CRUNCH) framework (Reuter-Lorenz and Cappell, 2008), lower efficiency in neural processing may lead to increased recruitment of neural resources (i.e., over-activation). Therefore, the patients with CLBP had low efficiency in upright stance control due to pain, which might require higher DLPFC activation. The results of correlation analysis showed that there were relationships between DLPFC activation and COP parameters when sensory input was manipulated. Teo et al. (2018) also found that there was a significant positive correlation between DLPFC activation and balance performance in healthy adults, especially under conditions with greater sensory restriction. Previous studies have confirmed that the DLPFC could help maintain postural stability by effectively allocating attention resources (Fujita et al., 2016; Teo et al., 2018) and planning motor sequences (Kaller et al., 2011) when performing motor tasks. Although our results did not show a significant condition main effect in DLPFC, the results of the *t*-test exhibited that the patients with CLBP showed right DLPFC significantly activated in Task-2 and bilateral DLPFC significantly activated in Task-3, and no cortex significantly activated in Task-1. These findings suggested that DLPFC played an important role in motor control in the patients with CLBP, which was required in balance tasks with limited sensory input rather than simple balance task. However, the patients with CLBP might have a low neural processing efficiency of attentional allocation, which was similar to the elderly people. Therefore, the CLBP patients required more cortical activity for functional compensation than the healthy controls. This was the potential reason for higher DLPFC activation of CLBP participants in the present study.

It is well-known that the PMC/SMA is involved in learning and planning postural control and balance recovery (Park et al., 2010; de la Peña et al., 2020). Previous studies have also confirmed that stable posture control is associated with cortical activity in the SMA (Fujita et al., 2016; Herold et al., 2017a; Kumai et al., 2022). In healthy controls, we observed that bilateral SMA activation was higher in Task-2 and Task-3 than in Task-1. When manipulating sensory feedback, these tasks may provide a new learning experience for two groups of participants. The PMC/SMA is involved in establishing new motor programs to maintain balance (Takakura et al., 2015). Our results also showed significant correlations between PMC/SMA activation and COP parameters in the healthy control group during static conditions. Our correlation results indicated that PMC/SMA plays an essential role in static balance. However, this was inconsistent with the findings in previous studies. Previous studies have found a correlation between SMA activation and ML sway acceleration in dynamic balance task (Herold et al., 2017a; Kumai et al., 2022). The potential reason was that we used different COP parameters in this study. However, no such condition effects and correlations were observed in the patients with CLBP, which may indicate that PMC/SMA in patients with CLBP has functional defects. Under Task-1, the patients with CLBP had significantly higher bilateral PMC/SMA activation than the healthy controls. Due to the low complexity of the task and low motor needs, the patients with CLBP may require more PMC/SMA activation to maintain balance compared with the healthy controls, which exhibited significant inverse oxygenation responses (Holper et al., 2011; Shi et al., 2021; Almulla et al., 2022). When visual feedback was absent, compared with healthy controls, the patients with CLBP had significantly lower left PMC/SMA activation accompanied by a larger sway area. This neural and behavioral pattern might indicate insufficient utilization of motor resources in patients with CLBP (Pelicioni et al., 2020). SMA also coordinates limb movements (Debaere et al., 2001). Increased PMC/SMA activation in dynamic conditions may reflect the preparation for muscle activity and joint movements to prevent falls (Hiyamizu et al., 2014; Fujita et al., 2016). M1 is a part of the direct locomotor pathway, which plays a dominant role in motor preparation and execution (Endo et al., 1999). There was a strong tendency toward statistical significance in the difference in right M1 activation between the two groups (*p* = 0.050). This result may indicate that the direct locomotor pathway in patients with CLBP may have a lower neural efficiency during upright stance. Although our results did not show significant group differences in S1 activation, we found a significant correlation between COP parameters and S1 activation in healthy controls when visual input was absent. This relationship may suggest that S1 needs to integrate all sensory information to maintain stability when vision is absent (Hooks, 2017). However, the patients with CLBP did not show such a relationship. The potential reason was that they had a changed function in S1, which Vartiainen et al. (2009) reported. Meanwhile, with the change in proprioceptive information inputs, left S1 activation increased. This result is consistent with a previous study by Herold et al. (2017a), which implied that S1 contributes to the integration of proprioception.

### Limitations

The present study has several limitations. First, due to the low temporal resolution of fNIRS, future studies should use EEG-combined fNIRS to explore neural mechanisms. Second, due to the relatively low spatial resolution of fNIRS, the calculated MNI coordinates might fall outside the brain or be not in the center of the brain region. Each channel often measures the mean activity changes of several brain regions with different overlap probabilities in this channel. In this study, the selected channel only represented a part of the brain region with the highest overlap probabilities, which is prone to decrease the robustness of the statistics in the present study. Moreover, a relevant concern is owing to the fact the fNIRS was applied on the upper limb representation of M1, while behavior changes were mostly observed in balance functions. Therefore, it’s necessary to measure M1 activation for trunk and lower limb representation. In the future study, it would be better to increase the number of sources and detectors to obtain the best representative channel for the ROIs. Third, dual-task might be a better experimental design to show the roles of DLPFC in attention and upright stance control, respectively. Unfortunately, the present study did not employ the dual-task design. It would hide the interpretation of the role of DLPFC in upright stance control within the context in which DLPFC was also required in attentional control. Future studies should employ the dual-task design to explain the roles of DLPFC in attention and upright stance control in patients with CLBP. Finally, we only monitored the change in COP displacement as a behavioral measurement and lacked muscle activity and kinematics. Therefore, we could not assess the relationship between cortical activation, muscle activity, and kinematics.

## Conclusion

Consistent with previous findings, the patients with CLBP showed uptight stance control dysfunctions. We explored the difference in the activity of the cognitive and sensorimotor cortices between the patients with CLBP and healthy people under different upright stance tasks. Results imply that DLPFC and PMC/SMA play a role in upright stance control. The patients with CLBP had increased right DLPFC activation and altered bilateral PMC/SMA activation during upright stance. The upright stance control deficits of the patients with CLBP may be related to the change of cortical function, especially in DLPFC. It helped us clarify the neural mechanisms underlying upright stance control dysfunction in patients with CLBP. Future studies should explore whether this increased cortical activity in patients with CLBP is modified after rehabilitation.

## Data availability statement

The raw data supporting the conclusions of this article will be made available by the authors, without undue reservation.

## Ethics statement

The studies involving human participants were reviewed and approved by Ethical Committee for Human Experiments of the First Affiliated Hospital of Sun Yat-sen University. The patients/participants provided their written informed consent to participate in this study.

## Author contributions

YL, ZX, HX, RF, WL, XC, JY, LG, QY, and CW worked together to complete the manuscript. YL and ZX designed the methodology and drafted the manuscript for the study. ZX, HX, and RF participated in the data acquisition and data curation. XC, JY, and LG analyzed and interpreted the data. WL, QY, and CW critically revised the manuscript for content. All authors contributed to the article and approved the submitted version.
